# Identification of novel genes involved in the biofilm formation process of Avian Pathogenic *Escherichia coli* (APEC)

**DOI:** 10.1371/journal.pone.0279206

**Published:** 2022-12-19

**Authors:** Meaghan M. Young, Aline L. de Oliveira, Lisa K. Nolan, Nicolle L. Barbieri, Catherine M. Logue

**Affiliations:** 1 Department of Population Health, College of Veterinary Medicine, University of Georgia, Athens, Georgia, United States of America; 2 Department Microbiology, Franklin College of Arts and Sciences, University of Georgia, Athens, Georgia, United States of America; 3 Department of Infectious Diseases, College of Veterinary Medicine, University of Georgia, Athens, Georgia, United States of America; USDA-ARS Eastern Regional Research Center, UNITED STATES

## Abstract

Avian pathogenic *Escherichia coli* (APEC) is the etiological agent of avian colibacillosis, a leading cause of economic loss to the poultry industry worldwide. APEC causes disease using a diverse repertoire of virulence factors and has the ability to form biofilms, which contributes to the survival and persistence of APEC in harsh environments. The objective of this study was to identify genes most widespread and important in APEC that contribute to APEC biofilm formation. Using the characterized APEC O18 as the template strain, a total of 15,660 mutants were randomly generated using signature tagged mutagenesis and evaluated for decreased biofilm formation ability using the crystal violet assay. Biofilm deficient mutants were sequenced, and a total of 547 putative biofilm formation genes were identified. Thirty of these genes were analyzed by PCR for prevalence among 109 APEC isolates and 104 avian fecal *E*. *coli* (AFEC) isolates, resulting in nine genes with significantly greater prevalence in APEC than AFEC. The expression of these genes was evaluated in the wild-type APEC O18 strain using quantitative real-time PCR (qPCR) in both the exponential growth phase and the mature biofilm phase. To investigate the role of these genes in biofilm formation, isogenic mutants were constructed and evaluated for their biofilm production and planktonic growth abilities. Four of the mutants (*rfaY*, *rfaI*, and two uncharacterized genes) displayed significantly decreased biofilm formation, and of those four, one (*rfaI*) displayed significantly decreased growth compared to the wild type. Overall, this study identified novel genes that may be important in APEC and its biofilm formation. The data generated from this study will benefit further investigation into the mechanisms of APEC biofilm formation.

## Introduction

Avian pathogenic *Escherichia coli* (APEC) is an extraintestinal pathotype of *E*. *coli* that causes disease in poultry and other avian species. It the etiological agent of avian colibacillosis, a leading bacterial cause of morbidity and mortality in the poultry industry that contributes to significant economic loss worldwide each year [1]. There are many virulence factors associated with APEC, including those that contribute to the ability to persist in the environment, resist antimicrobials, and acquire genetic information from other microbes [2–4]. Biofilm formation is a major contributing factor to the survival and persistence of APEC, and research into the genetic makeup of biofilm is a growing field [5–7].

Biofilms form when planktonic microorganisms attach to a surface and secrete exopolysaccharides, proteins, and nucleic acids to form the extracellular biofilm matrix [8]. Biofilm formation can be divided into three main stages: (1) attachment to a surface, (2) aggregation and formation of mature biofilm architecture, and (3) dispersion from structure [9]. Biofilm formation is a response to stressful conditions and serves as a physical barrier protecting against harmful environmental factors, including antimicrobials, host defenses, and predation [10,11]. Biofilms also provide a safe environment for horizontal gene transfer and increase conjugation efficiency between bacterial cells, which can lead to increased transfer of antimicrobial resistance and other virulence genes [12,13].

Research into the role and function of APEC biofilm formation process is ongoing. Factors associated with biofilm include the type I fimbriae, encoded by genes on the *fim* operon, and curli proteins, encoded by genes on two *csg* operons, which are involved in the initial attachment of planktonic cells to a surface and in cell-to-cell adhesion [14,15]. Flagella, and in turn motility, have been found to play a role in the adhesion of *E*. *coli* to abiotic surfaces and in influencing the biofilm architecture [16,17]. Outer membrane proteins have also been implicated in a variety of roles in biofilm formation, from cell-to-cell communication to mature biofilm development [18–20]. In addition, quorum sensing has been implicated in multiple aspects of biofilm formation and development, from structural integrity to cellular dispersal [21]. Despite these findings, there is still limited knowledge on which genes may be linked to biofilm formation in APEC specifically. By discovering new genes associated with the biofilm formation of APEC, we may better characterize the virulence profile and biofilm formation ability of APEC.

The purpose of this study is to uncover new genes involved in the biofilm formation process of APEC O18, a well-characterized and sequenced APEC strain, and to determine if they are widespread and important in APEC. This study was able to identify 547 biofilm genes, 30 of which were more prevalent in APEC isolates than avian fecal isolates, indicating that these genes may be viable APEC targets. Nine of the APEC genes were selected for further analysis, resulting in seven of the genes appearing to contribute to APEC biofilm formation. To the author’s knowledge, this is the first study to definitively identify genes involved in the biofilm formation process that are specific to APEC.

## Materials and methods

### Bacterial strains, plasmids, and growth conditions

Strains and plasmids are shown in Table 1. The wild-type template in this study was APEC O18 (denoted as AO18 WT), which is a virulent O-serogroup 18, sequence type 95, and phylogenetic group B2 strain of *E*. *coli*. The strain was isolated from the pericardial and lung tissue of a laying hen diagnosed with colibacillosis in the United States and is a robust biofilm producer. The genome of APEC O18 was sequenced and is publicly available in GenBank (accession number CP006830) under the name “APEC O18” [22]. *E*. *coli* DH5α was used for plasmid cloning. All strains were cultured in Luria-Bertani (LB) broth, Miller (BD Difco™, Franklin Lakes, NJ) at 37°C with agitation unless otherwise specified. The medium was supplemented with ampicillin (AMP, 100 mg/mL), kanamycin (KAN, 50 mg/mL), nalidixic acid (NAL, 30 mg/mL), or chloramphenicol (CHM, 25 μg/mL) as necessary.

**Table 1:** Strains and plasmids in this study.

**Plasmid**	**Genotype/Description**	**Reference**
pUTmini-Tn5*km2*	Insertion mutagenesis transposon	[23]
pKD46	λ Red recombinase expression plasmid	[24]
pKD3	Template plasmid for FRT-flanked CHM resistance cassette	[24]
pCP20	Temperature-sensitive FLP recombinase expression plasmid	[24]
pACYC184	Cloning vector	[25]
pACYC184-*rfaY*	pACYC184 with *rfaY*	This study
pACYC184-*nanM*	pACYC184 with *nanM*	This study
pACYC184-*nhaC*	pACYC184 with *nhaC*	This study
pACYC184*-rfaI*	pACYC184 with *rfaI*	This study
pACYC184-*hypo01*	pACYC184 with *hypo01*	This study
pACYC184-*abh*	pACYC184 with *abh*	This study
pACYC184-*hypo11*	pACYC184 with *hypo11*	This study
pACYC184-*hypo14*	pACYC184 with *hypo14*	This study
**Strain**	**Genotype/Description**	**Reference**
AO18 WT	Wild-type template	Lab stock
*E*. *coli* S17-1 λpir	Donor strain for conjugation	[23]
DH5α	Plasmid cloning vessel	Lab stock
AO18Δ*rfaY*-T	AO18 with *rfaY* disruption by pUTmini-Tn5*km2* transposon insertion	This study
AO18Δ*rfaY*	AO18 with *rfaY* deletion by λ red recombinase	This study
AO18Δ*rfaY*-C	AO18Δ*rfaY* with pACYC184-*rfaY*	This study
AO18Δ*nanM*-T	AO18 with *nanM* disruption by pUTmini-Tn5*km2* transposon insertion	This study
AO18Δ*nanM*	AO18 with *nanM* deletion by λ red recombinase	This study
AO18Δ*nanM*-C	AO18Δ*nanM* with pACYC184-*nanM*	This study
AO18Δ*nhaC*-T	AO18 with *nhaC* disruption by pUTmini-Tn5*km2* transposon insertion	This study
AO18Δ*nhaC*	AO18 with *nhaC* deletion by λ red recombinase	This study
AO18Δ*nhaC*-C	AO18Δ*nhaC* with pACYC184-*nhaC*	This study
AO18Δ*rfaI*-T	AO18 with *rfaI* disruption by pUTmini-Tn5*km*2 transposon insertion	This study
AO18Δ*rfaI*	AO18 with *rfaI* deletion by λ red recombinase	This study
AO18Δ*rfaI*-C	AO18Δ*rfaI* with pACYC184*-rfaI*	This study
AO18Δ*hypo01*-T	AO18 with *hypo01* disruption by pUTmini-Tn5*km2* transposon insertion	This study
AO18Δ*hypo01*	AO18 with *hypo01* deletion by λ red recombinase	This study
AO18Δ*hypo01*-C	AO18Δ*hypo01* with pACYC184-*hypo01*	This study
AO18Δ*abh*-T	AO18 with *abh* disruption by pUTmini-Tn5*km2* transposon insertion	This study
AO18Δ*abh*	AO18 with *abh* deletion by λ red recombinase	This study
AO18Δ*abh*-C	AO18Δ*abh* with pACYC184-*abh*	This study
AO18Δ*hypo11*-T	AO18 with *hypo11* disruption by pUTmini-Tn5*km2* transposon insertion	This study
AO18Δ*hypo11*	AO18 with *hypo11* deletion by λ red recombinase	This study
AO18Δ*hypo11*-C	AO18Δ*hypo11* with pACYC184-*hypo11*	This study
AO18Δ*hypo14*-T	AO18 with *hypo14* disruption by pUTmini-Tn5*km2* transposon insertion	This study
AO18Δ*hypo14*	AO18 with *hypo14* deletion by λ red recombinase	This study
AO18Δ*hypo14*-C	AO18Δ*hypo14* with pACYC184-*hypo14*	This study

### DNA extraction

Bacterial DNA was extracted from strains by boiling. Briefly, strains were streaked out from frozen stocks on Luria-Bertani (LB) agar, Miller (BD Difco™, Franklin Lakes, NJ, USA) and incubated overnight at 37°C. Following incubation, a 1-μL inoculating loop was used to collect bacteria from the plate and inoculate 200 μL of sterile Milli-Q water. The mixture was boiled for 10 min at 99°C and then centrifuged for 3 min at 13,000 x g to precipitate cellular debris. A volume of 150 μL of the supernatant containing the genomic material was transferred to a fresh tube and used as the DNA template for gene amplification. The bacterial DNA stocks were stored at -20°C until use.

### Mutant library generation

A random mutant library was generated using the signature tagged mutagenesis (STM) technique using transposon pUTmini-Tn5km2, as previously described [23]. Independent matings were set by growing each *E*. *coli* S17-1 λpir (with tag) clone and the APEC O18 nalidixic acid-resistant clone to the late log phase. To construct tagged transposon mutants of APEC O18, 400 μL of donor cells and 400 μL of recipient cells were mixed and incubated at 37°C for 8 h on plates. Cells were collected from the plates and re-suspended in phosphate buffered saline (PBS; Research Products International, Mt. Prospect, IL) and plated onto LB agar containing 50 mg/mL KAN and 30 mg/mL NAL (NAL-KAN-LB). Following selective overnight growth, single colonies were selected and inoculated into 1-mL wells of 96-well deepwell microtiter plates (Eppendorf, Hamburg, Germany). Glycerol was added to a final concentration of 15–20%, and the plates sealed and stored at -80°C.

### Biofilm assays for impaired biofilm formation

The mutant libraries were tested for biofilm formation abilities in M63 minimal medium, as previously described [6]. Briefly, mutants were grown overnight in LB broth. Following incubation, the mutants were diluted 1:100 in M63 minimal medium [12 g KH_2_PO_4_ per liter, 28 g K_2_HPO_4_ per liter, 8 g (NH_4_)_2_SO_4_ per liter, supplemented with 1 mmol per liter of MgSO_4_, 0.2% glucose, and 0.5% casamino acids]. Aliquots of 200 μL of each dilution were dispensed into the wells of a 96-well microtiter plate (Sarstedt AG & Co. KG, Sarstedt, Germany). Negative control wells contained uninoculated medium, and positive control wells were inoculated with AO18 WT. Each mutant was tested once. Following static incubation at 37°C for 24 h, the contents of the plates were poured off and the wells of the plate washed once with sterile Milli-Q water. Next, the wells were stained with 200 μL of 0.1% crystal violet solution (Fisher Scientific Company, Fair Lawn, NJ) for 30 min, washed four times with Milli-Q water to remove excess stain, and air dried for 1 h. After drying, adherent cells were re-solubilized with 200 μL of an 80:20 solution of ethanol and acetone (Fisher Scientific Company, Fair Lawn, NJ). A volume of 150 μL of this solution was transferred to a new microtiter plate, and the optical density of each well was measured at 600 nm (OD_600_) using an automated ELx808 Ultra MicroPlate Reader (Bio-Tek Instruments, Winooski, VT). All analyses were carried out in triplicate, and the results were averaged [6].

Mutants with an OD_600_ less than 50% of the positive control were tested a second time to confirm low biofilm production. Mutants that produced less than 50% of the positive control a second time were selected for further analysis.

### Mutant sequence analysis

Mutants were sequenced according to the primers and protocol described by [23]. Briefly, selected mutants were cultured on NAL-KAN-LB agar plates and incubated overnight at 37°C. PCR was first done to amplify the transposon sites using Arbi5 in combination with P9, a transposon-specific primer. Next, a second round of nested PCR was performed using 1 μL of each original PCR product. Arbi2 was used, as it is homologous to the 5’ sequence of Arbi5, and P6 was used as a transposon I terminus-specific primer [23]. The second round of PCR products generated were purified using ExoSAP to remove primer and sent for Sanger sequencing to the Iowa State University sequencing facilities.

Data analysis of the sequenced PCR products was done using the Nucleotide Basic Local Alignment Search Tool (BLASTN) algorithms on public databases [26,27]. A literature analysis was performed on the putative biofilm formation genes identified by BLASTN analysis using PubMed and Google Scholar. The 30 most common "hits” that have not yet been characterized in APEC biofilm formation were selected for further analysis.

### Prevalence analysis

The prevalence of 30 selected putative biofilm formation genes was determined in a sample of 109 APEC and 104 avian fecal *E*. *coli* (AFEC) isolates collected from previous studies [28]. AFEC isolates were compared to APEC strains to identify genes more prevalent in the pathogenic strains (APEC) than the commensal fecal strains (AFEC). Genes were selected if they were frequently found among the transposon mutants and if they did not have a known role in APEC-specific biofilm formation. The detection of genes was analyzed using six multiplex PCR panels. Primers used are detailed in Table 2. Multiplex primers were created by combining equal volumes of each primer (starting concentration of 100 μM) listed. The primers for each gene were designed using AO18 WT as the template with Primer3 (v0.4.0), and the multiplex panels were constructed using Geneious Prime (Version 2021.1.1). PCR was performed using 2 μL DNA, 2.5 μL PCR buffer (10x), 0.4 μL MgCl_2_ (50 mM), 1.25 μL dNTP mixture (10μM), 2.0 U Taq DNA polymerase, 1.0 μL of multiplex primer, and 15.85 μL sterile Milli-Q water. The thermal cycle conditions consisted of a 5-minute activation step at 94°C, followed by 30 annealing cycles of 94°C for 30 s, 54–59°C for 30 s (depending on multiplex), and 72°C for 3 min, and a final extension step of 72°C for 10 min. The annealing temperate for multiplex 1, 2, and 3 was 55°C, for multiplex 4 was 59°C, and for multiplex 5 and 6 was 54°C. The amplicons were then separated by 1.5% agarose gel electrophoresis (MSP brand Agarose LE, Atlanta, GA), stained with 0.1% ethidium bromide (Sigma Aldrich, St. Louis, MO), and visualized under UV light using a UVP GelSolo (Analytik Jena, Jena, Germany).

**Table 2:** Primers used in this study.

Primer	Sequence (5’ - 3’)
	**Prevalence Analysis**
rfaY_F1^a^	TTAAAGTCTTTGCCCCGAAA
rfaY_R1	AGGCATGCAATTTTTCCATT
kbaY_F1	CCTCGAAGTGTGCAGTGAAA
kbaY_R1	CTTGTGGATCGGTCAGGAAT
nanM_F1	CCGAGAGATCAAGCAACCTC
nanM_R1	CCCCTCCGGCAAATATAAGT
nhaC_F1	CATGCCGATCTTCCAATTCT
nhaC_R1	ATGGTGCCAAAATCTTCCAG
yjeM_F1	CTCCACCTGGGTTTCTACCA
yjeM_R1	TGTATTTCCACCACGCAAAG
xylF_F2	CGATGCGGATATCGATTTTT
xylF_R2	GGTTAGCGCGTTTTCCATAA
waaV_F2	AGGGGATGCGATTTATTGTG
waaV_R2	AGATTCTTTCGTGCGCAAAT
rfaI_F2	GCTTAAGGCATTACCGACGA
rfaI_R2	ATAAAAGGTTGCGCACTTGG
gatC_F2	TTAACGTGGCGATGCTACTG
gatC_R2	TCCACAGGGTGATGCTCATA
yliE_F2	AGGCGATGCGAAAGAGATTA
yliE_R2	CCCCTTGTTTACGCAGTTGT
hypo02_F3	GGCATGAAAGAACTTGCTGA
hypo02_R3	GGTCAAGCGATGACCATTAAA
msyB_F3	GATGCGCAACGAACTGTTTA
msyB_R3	CAAATGCGACACACCTTTTG
rfaJ_F3	TTTTGGTCGTACGCGACATA
rfaJ_R3	TGTTCACGCGCGATATTAAA
hypo01_F3	CGTATCCCTGTGCACCTTCT
hypo01_R3	TGTGGTCATCAGGCTTTCTG
hypo03_F3	GCAGGTGAAAACGGAGATGT
hypo03_R3	CATTCATAACGCCAAGTTCG
bioD_F4	CCTTACACCTTCGCAGAACC
bioD_R4	AGTCCGGCGTGTAGTATTGC
sohB_F4	GCGTCTGCGTGATAAAAACA
sohB_R4	TTTCATCAACCAGGCCTTTC
dhaK_F4	CACACCAGACCAAATGATCG
dhaK_R4	GTGCTGCCAAGTCCGTTAAT
yicL_F4	GCTGTGGCAGTGGGATAAAT
yicL_R4	AAAGATCACCGAACCGTCAG
abh_F4	TGGTGAGGAAAAACCGTCTC
abh_R4	ATTAATCCAGGGCGTTGTTG
hypo06_F5	GGGAAAACCAAATCCCTGAT
hypo06_R5	CGTCGCGTATGAACCTGTAA
hypo11_F5	AAACTGGCGTGAGGATGAAC
hypo11_R5	CATCGCGCATAAACACTCAT
hypo13_F5	TGGCCTACTTTCAGGCGTAT
hypo13_R5	GCGTATGGCGAATCATTTCT
hypo12_F5	TACTTCCTTTGTCCCGGTTG
hypo12_R5	GCGCTCTGATTTGTTCCTTC
hypo05_F5	TGCTTTCCGAGTCTGTTCCT
hypo05_R5	GCGTAATCCAGTTTCCGTGT
hypo09_F6	TCAGCCAGAAAAATGTGGTG
hypo09_R6	CGCTGACTGTCTGACCAAAA
hypo14_F6	CGAAAATGCGCTCAATGTTA
hypo14_R6	TTTCCCCCAACTTTTTACCC
hypo08_F6	TAAAAACGTTCCCCAGCAAC
hypo08_R6	TCATAATTCACAGGCGACCA
hypo07_F6	TGCACCCGATCTCAATATCA
hypo07_R6	GCTTCTGCATCGCAATGTTA
hypo10_F6	AAAGCCGCACTTGACCTTTA
hypo10_R6	CGACCAGCGATAATCACCTT
	**Gene Deletion**
rfaY_MUT_F	ATGATTACAAGTATACGCTATCGCGGCTTCTCATTTTATTACAAAGATAAtgtaggctggagctgcttcg^b^
rfaY_MUT_R	TTACGCTTTGCCTTTTAATTTTTTAATAAATTTGCGTAGTTTGGTTCTGTatgggaattagccatggtcc^c^
nanM_MUT_F	ATGAATAAAACAATAACGGCGCTTACTATCATAATGGCTTCATTTGCCACtgtaggctggagctgcttcg
nanM_MUT_R	TTAGTTTTGTACTGTGACTTTATTATCCTTCACAGAAATCAAAACTGAATatgggaattagccatggtcc
rfaI_MUT_F	ATGAGTGCCCACTATTTTAATCCACAAGAGATGATCAATAAGACAATCATtgtaggctggagctgcttcg
rfaI_MUT_R	TCATTTTATTATCTTTAAATAAAAATAATAAATATAATTCATTATCCCGTatgggaattagccatggtcc
nhaC_MUT_F	ATGAGAGAAAAACCCAGTTTTTATGTCGCGCTTACACCGATCATTTTTATtgtaggctggagctgcttcg
nhaC_MUT_R	TCAGGCCTTTGCTTCGTTGAAGCGCAGTAAACGGAAACCTGTAGAAGCATatgggaattagccatggtcc
hypo01_MUT_F	ATGAAAACGAATAATGCCGGTTATATTATCGGCGCGTATCCCTGTGCACCtgtaggctggagctgcttcg
hypo01_MUT_R	TTAACGAGATTCATTCAGAGAGTGAATGCCGTTGCGTAATATTTCGATGCatgggaattagccatggtcc
abh_MUT_F	ATGCGAAAAATAATTACTCATTTCAAAGTTGTTTTAACGTTACTTCTACCtgtaggctggagctgcttcg
abh_MUT_R	TTATTGTATTTCTGCTTCAGAATTTCCTGGGCAGTATATATTTTCTGGTTatgggaattagccatggtcc
hypo11_MUT_F	ATGCCCCCCACTCCTGCCATGCAGGCATTAATTGAGCAGATATATCATATtgtaggctggagctgcttcg
hypo11_MUT_R	TTATTTTCCTGGTGATTCGGATGATGCGTCATACATCGCGCATAAACACTatgggaattagccatggtcc
hypo14_MUT_F	ATGAAATCCGAGACGCTAACTGTCCAACAACTTTTTCAAGACCGCCGACAtgtaggctggagctgcttcg
hypo14_MUT_R	TTAATCGTGTTTTGGCCATACTTTCAATGCAATTTCCCCCAACTTTTTACatgggaattagccatggtcc
	**Check Deletion**
rfaY_check1_F	TTTAATATCGCGCGTGAACA
rfaY_check1_R	TGCCGCACCAAATAAAAAGT
nanM_check1_F	AGGGTGTTTACAACGGCAGA
nanM_check1_R	GCATCTTCCTTGTCCGGTAA
rfaI_check1_F	AGGAACAAGGGCTGCTGTC
rfaI_check1_R	CTCTGGGGCGTTTTCTTTTT
nhaC_check1_F	TGTCACTGGAGAAGGTGCAG
nhaC_check1_R	CGAGCTGGAGATTGTGCTTA
hypo01_check1_F	CACTACGGCAATACGCAAGA
hypo01_check1_R	GCCTTCGAGTATCCGTTTCA
abh_check1_F	GATTTTCTCCCCGGTGGTAT
abh_check1_R	TGGCACGTCAACTTTTGATT
hypo11_check1_F	GAGGAAAGCTGTTGGGACTG
hypo11_check1_R	CAGGAGCGGAAAGGAGAATA
hypo14_check1_F	CCGATGGGATGATGAAGAGA
hypo14_check1_R	ACGTAATGCGCTGAACTGTG
	**Gene Complementation**
rfaY_comp_F	TACGGATCCACATGATTACAAGTATACGCTA ^d^
rfaY_comp_R	TAAAGTCGACACTTACGCTTTGCCTTTTAATT ^e^
nanM_comp_F	TACGGATCCACATGAATAAAACAATAACGGC
nanM_comp_R	TAAAGTCGACACTTAGTTTTGTACTGTGACTT
rfaI_comp_F	TACGGATCCACATGAGTGCCCACTATTTTAA
rfaI_comp_R	TAAAGTCGACACTCATTTTATTATCTTTAAATAAAAATAA
nhaC_comp_F	TACGGATCCACATGAGAGAAAAACCCAGTTT
nhaC_comp_R	TAAAGTCGACACTCAGGCCTTTGCTTCGTTGA
hypo01_comp_F	TACGGATCCACATGAAAACGAATAATGCCGG
hypo01_comp_R	TAAAGTCGACACTTAACGAGATTCATTCAGAG
abh_comp_F	TACGGATCCACATGCGAAAAATAATTACTCA
abh_comp_R	TAAAGTCGACACTTATTGTATTTCTGCTTCAG
hypo11_comp_F	TACGGATCCACATGCCCCCCACTCCTG
hypo11_comp_R	TAAAGTCGACACTTATTTTCCTGGTGATTCGGATGATGCG
hypo14_comp_F	TACGGATCCACATGAAATCCGAGACGCTAACTGT
hypo14_comp_R	TAAAGTCGACACTTAATCGTGTTTTGGCCATACTTTCAATGC
	**Check Complementation**
pACYC_F	CGACCACACCCGTCCTGT
pACYC_R	AAGGCTCTCAAGGGCATCG
	**qPCR**
rfaY_RT_F	CCGAAAACGAAAAGGAATGA
rfaY_RT_R	AGCTCTACGCCCTCGACATA
nanM_RT_F	GGCGGTTGTGAATAAAGGTG
nanM_RT_R	CCCCTCCGGCAAATATAAGT
rfaI_RT_F	TGCCATGTATTTCCGTTTTG
rfaI_RT_R	TCGCGTTCAGTAACAACAGC
nhaC_RT_F	ATGCCGATCTTCCAATTCTG
nhaC_RT_R	CATTCCCAGCCAGGTAAAAA
hypo01_RT_F	CGTATCCCTGTGCACCTTCT
hypo01_RT_R	TTTCCAGCTCTCTGGCGTAT
abh_RT_F	GACCTTCTGCTATGGCGTTC
abh_RT_R	TGCTCATCCCCCTGATCTAC
hypo11_RT_F	TTGCGAGCGATTCCTTATTC
hypo11_RT_R	TCCTCACGCCAGTTTTCTTT
hypo14_RT_F	CATTTTAATCACCCGCCATC
hypo14_RT_R	ATTTGGGCATCATCTTCAGC

^a^Number indicates which multiplex panel primer is in (1, 2, 3, 4, 5, or 6).

^b^Forward primer extension to amplify chloramphenicol resistance cassette (tgtaggctggagctgcttcg).

^c^Reverse primer extension to amplify chloramphenicol resistance cassette (atgggaattagccatggtcc).

^d^Forward primer extension to amplify BamHI restriction site (GGATCC).

^e^Reverse primer extension to amplify SalI restriction site (GTCGAC).

### *In silico* prevalence analysis

The prevalence of the 30 genes was also analyzed with *in silico* PCR using Geneious Prime. The genomes of 11 APEC, six human extraintestinal pathogenic (ExPEC), three intestinal pathogenic, one fecal *E*. *coli*, and nine laboratory strains of *E*. *coli* were imported from NCBI into Geneious Prime. Primers used were the same sequences as those detailed in Table 2 for the prevalence analysis.

### Construction of mutants and complemented strains

Isogenic mutants were constructed using λ red mutagenesis [24]. Primers for mutant construction and complementation are detailed in Table 2. Briefly, oligonucleotides specific to the CHM cassette flanked by 50 nucleotide extensions homologous to the 5’ and 3’ of the gene to be deleted were used to amplify the CHM resistance cassette from plasmid pKD3 (ATCC®, Manassas, VA). The PCR products were run on a 1.5% agarose gel, and the DNA of the resulting fragment was extracted from the gel using QIAquick Gel Extraction Kit (Qiagen, Germantown, MD). The resulting DNA fragments were electroporated into APEC O18 containing the λ red recombinase plasmid pKD46 (ATCC®, Manassas, VA). After electroporation, the cells were grown in super optimal broth with catabolite expression (SOC) for 90 min at 37°C and plated on LB agar containing 25 mg/mL CHM (CHM-LB). Colonies were screened by PCR to identify deletion mutants and then confirmed using Sanger sequencing (Eurofins Genomics LLC, Louisville, KY). The CHM cassettes were cured from the strains by transforming helper plasmid pCP20 (ATCC®, Manassas, VA) into the mutants and confirmed both phenotypically by screening for CHM-sensitive colonies and genotypically using PCR.

Complementation was performed as previously described with modifications [25]. Briefly, PCR-amplified genes of interest were cloned into the BamHI and SalI restriction sites of plasmid pACYC184 (New England Biolabs, Ipswich, MA). The cloned plasmids were confirmed via Sanger sequencing, and then electroporated into their mutant counterparts. After electroporation, the cells were grown in SOC for 90 min at 37°C and plated on CHM-LB agar. Complements were screened via PCR and confirmed via Sanger sequencing.

### Growth curve assays

The growth rates of the AO18 WT and mutant strains in M63 minimal medium were analyzed. Briefly, strains were incubated statically overnight in LB broth with or without antibiotics at 37°C as indicated above. The overnight cultures were diluted 1:100 in LB broth with or without antimicrobials, then grown until the exponential growth phase (OD_600_ = ~0.5–0.6). Next, the OD_600_ of the cultures was measured using an Implen NanoPhotometer® NP80 (Implen, Munich, Germany), and cultures were diluted 1:100 in M63 minimal medium. Aliquots of 200 μL were dispensed into the wells of a 96-well microtiter plate and incubated with shaking for 12 h at 37°C. Negative control wells contained uninoculated medium, and the positive control wells were inoculated with AO18 WT. The OD_595_ was measured every 10 min using a Multiskan™ FC Microplate Photometer with incubator (Thermo Scientific™, Waltham, MA). Growth curves were performed with eight technical replicates on three separate days, and the absorbance data were averaged and plotted against time to build the growth curves.

### Biofilm formation assays for mutants

The biofilm formation of AO18 WT and mutant strains was analyzed as previously described [6] and detailed above. Biofilm assays were performed with eight technical replicates on three separate days, and the absorbance data were averaged.

### Expression of biofilm genes

#### Planktonic RNA extraction

RNA was extracted from planktonic AO18 WT cells using the RiboPure™-Bacteria RNA purification kit from Ambion™ (Austin, TX). Briefly, AO18 WT was grown overnight at 37°C in LB broth. The overnight cultures were diluted 1:100 in M63 minimal medium and grown at 37°C until the exponential phase. RNA was extracted from each culture according to the manufacturer’s instructions. Isolated RNA was treated with DNase I according to the manufacturer’s instructions to eliminate genomic DNA. The concentration and quality of RNA was measured using an Implen NanoPhotometer® NP80, and the samples were stored at -80°C until use.

#### Biofilm RNA extraction

RNA was extracted from attached AO18 WT biofilm cells using the RiboPure™-Bacteria RNA purification kit from Ambion™. Briefly, AO18 WT was grown overnight at 37°C in LB broth. The overnight culture was diluted 1:100 in M63 minimal medium. Aliquots of 2 mL were dispensed into the wells of a 6-well non-treated multidish (Thermo Scientific Nunc™, Roskilde, Denmark) and incubated statically for 24 h at 37°C. Following incubation, the contents of the plates were poured off and the wells of the plate washed three times with PBS. The wells were then scraped with a cell scraper and re-suspended in 1 mL of PBS. A total of five wells per plate were pooled together for the experiment. RNA was extracted, treated, measured, and stored as above.

#### Synthesis of first strand of cDNA

DNase-treated RNA was reverse-transcribed using the First-Strand cDNA Synthesis Kit from APExBio (Boston, MA). Briefly, 1 μg of DNase-treated RNA was mixed with 1 μL Random Primers (50 μM) and 1 μL dNTP mixture (10 mM) and adjusted to 10 μL with the provided RNase-free water. To denature the RNA, the mixture was heated at 65°C for 5 min and then chilled on ice for 2 min. The reverse transcription reaction system was prepared by adding 4 μL First-Strand Buffer (5x), 1 μL RNase Inhibitor, Murine (40 U/μL), 1 μL Reverse Transcriptase (200 U/μL), and 4 μL RNase-free water to the denatured RNA mixture, for a total volume of 20 μL. The thermocycler conditions for cDNA synthesis were as follows: 25°C for 2 min, 42°C for 50 min, and 75°C for 15 min. The resulting cDNA templates were stored at -20°C until use.

#### Quantitative Real-Time PCR (qPCR)

Gene expression was analyzed by quantitative real-time PCR (qPCR) in a qTower3 G qPCR System (Analytik Jena, Jena, Germany) and analyzed using qPCRsoft (v4.1) software, as previously described [29]. Reactions were performed using SYBR® Green (GoldBio, St. Louis, MO) according to the manufacturer’s instructions. Primers (Table 2) were created using Geneious Prime. Each gene was analyzed with biological and technical triplicates for planktonic RNA and biofilm RNA. Each reaction mixture contained 25 ng cDNA, 1 μL forward and reverse primers (2.5 μM), and 10 μL qPCR Master Mix (2x) with SYBR® Green and was adjusted to 20 μL with nuclease-free water (Ambion™, Austin, TX). The PCR conditions consisted of a 15-minute denaturation step at 95°C, followed by 40 annealing cycles of 95°C for 5 s and 60°C for 30 s. A melting curve analysis was performed at the end to ensure amplification specificity. Threshold fluorescence was established within the geometric phase of exponential amplification. The cycle threshold (CT) was determined for each sample by qPCRsoft and averaged among replicates. The housekeeping gene 16S rRNA was used as the endogenous control to normalize expression levels as previously described [29]. Differences in expression levels between the planktonic growth and biofilm maturation phases for each gene were calculated using the Livak method [30].

### Statistical analysis

For the prevalence analysis of genes harbored in the *E*. *coli* strains, the presence or absence of genes was treated as quantitative variables, and the Student’s t-test was used to evaluate the statistical significance (Microsoft Excel, Version 16.0). Biofilm formation was also evaluated using Student’s t-test in Excel. Gene expression Ct values were obtained using qPCRsoft and then transferred to Excel, where differences between planktonic expression and biofilm expression were analyzed using Student’s t-test. Growth curves were analyzed using linear regression to compare the exponential growth rate between strains (R Studio, Version 1.4.1106). All data from growth curves was included from when the bacterial population (OD_595_) had increased 150% from the inoculated concentration until the OD_595_ ceased to increase. Statistical significance for all tests was accepted when p < 0.05.

## Results

### Transposon library generation and analysis

A total of 15,660 mutants were randomly generated using the STM technique, which is three times the number of genes in the AO18 WT genome. This result gave 99% confidence that there was at least one mutant per gene in the genome. Of these mutants, 12,381 (79%) had no change in biofilm formation, 993 (6%) had increased biofilm formation (> 200% of the positive control), and 2,286 (15%) had decreased biofilm formation (< 50% of the positive control). The mutants with repeated decreased biofilm formation were sent for Sanger sequencing to identify where the disruption occurred.

Of the mutants sequenced, 920 mutant sequences were analyzed using BLASTN, resulting in 547 genes of interest. The functions of the identified genes, along with the count of genes, is displayed in S1 Fig. A literature analysis using PubMed and Google Scholar was completed for each gene to determine if they have been previously associated with the biofilm formation process of APEC. Thirty genes frequently identified among the transposon mutants and not known to be involved in the APEC-specific biofilm formation process were selected for prevalence analysis. The selected genes are described in S1 Table.

#### Prevalence analysis

**Fig 1:**
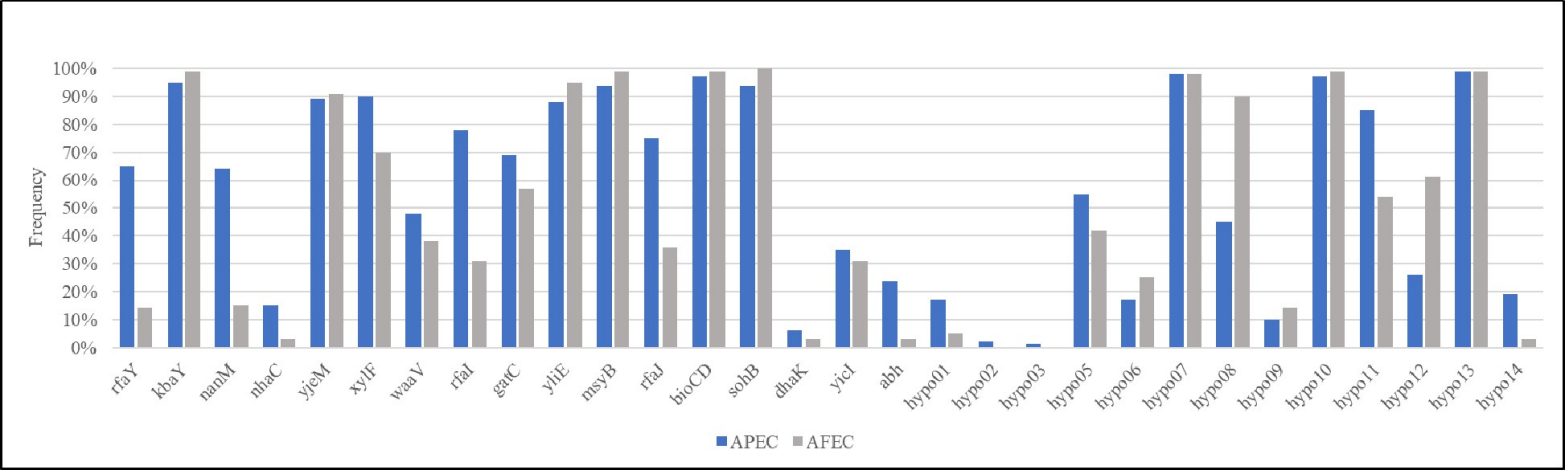
Prevalence analysis results. Thirty genes of interest were selected from the transposon mutant sequence analysis and analyzed for their prevalence using PCR in 109 clinical avian pathogenic *E*. *coli* (APEC) and 104 commensal avian fecal *E*. *coli* (AFEC) isolates. Asterisks (*) above brackets indicate significantly higher prevalence of that gene among the APEC isolates analyzed than the AFEC isolates (*p < 0.01).

In this study, 213 *E*. *coli* (109 APEC and 104 AFEC) isolates were screened for the presence of 30 putative biofilm formation genes. Fig 1 shows the overall prevalence of genes among the APEC and AFEC isolates.

The APEC isolates had a high prevalence (> 90%) of the following genes: *kbaY*, *xlyF*, *msyB*, *bioCD*, *sohB*, and hypothetical proteins *hypo07*, *hypo10*, and *hypo13*. These genes were also highly prevalent in the AFEC isolates, in addition to *yjeM*, *yliE*, and hypothetical protein *hypo08*.

The APEC isolates had a low prevalence (< 10%) of the following genes: *dhaK* and hypothetical proteins *hypo02* and *hypo03*. These genes were also low in prevalence among the AFEC isolates, in addition to *nhaC*, a putative alpha-beta hydrolase (represented here as *abh*), and hypothetical proteins *hypo01* and *hypo14*.

**Fig 2:**
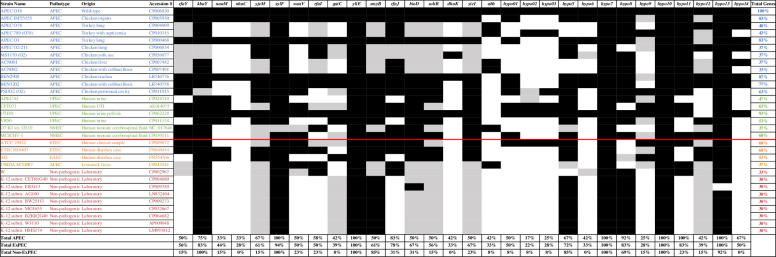
Results of *in silico* PCR on Geneious Prime. The prevalence of the thirty genes of interest in this study were evaluated in 31 strains of *E*. *coli* with *in silico* PCR using Geneious Prime. Strains evaluated were obtained from NCBI databases. Extraintestinal pathogenic *E*. *coli* (ExPEC) strains were compared to non-ExPEC strains, which are separated by the red line. ExPEC included 12 avian pathogenic *E*. *coli* (APEC, blue), four uropathogenic *E*. *coli* (UPEC, green), and two neonatal meningitis *E*. *coli* (NMEC, green). Non-ExPEC included two enterotoxigenic *E*. *coli* (ETEC, orange), one enteroaggregative *E*. *coli* (EAEC, orange), one livestock fecal *E*. *coli* (yellow), and nine laboratory strains of *E*. *coli* (red). Black boxes represent both forward and reverse primer sequences binding to the genome, gray represents only one sequence, and white represents no sequences. The “Total Genes” column is the percentage of genes present in the given strain, and the “Total” rows are the prevalence of each gene among the selected strains.

There were nine genes that displayed statistical and biological significance in prevalence in the APEC isolates compared to the AFEC: *rfaY*, *nanM*, *nhaC*, *rfaI*, *rfaJ*, *abh*, and hypothetical proteins *hypo01*, *hypo11*, and *hypo14* (p < 0.05). These results are supported by the *in silico* PCR analysis from Geneious Prime (Fig 2), in which all nine of these genes were more prevalent among the ExPEC isolates analyzed compared to the fecal isolates and K-12 strains. The prevalence of these genes in APEC more than AFEC indicates that these genes may be used to target APEC strains without disrupting the commensal *E*. *coli* population.

The lipopolysaccharide genes *rfaY* and *rfaI* of APEC O18 were aligned to those of all other *E*. *coli* strains analyzed in the *in silico* prevalence analysis using Geneious Prime. The APEC O18 *rfaY* and *rfaI* genes had 98.7–100% pairwise identity to 15 out of 17 ExPEC strains and 60.4% to two ExPEC strains. Four out of 13 non-ExPEC strains had 98.7–99.9% pairwise identity, and nine had 60.4–60.7% pairwise identity.

#### Growth curves

In order to determine if the selected genes affected the growth of APEC, the growth curves of AO18 WT, the isogenic mutants and complements, and their transposon mutant counterparts in M63 minimal medium were analyzed. The growth curve of each strain is displayed in S2 Fig. The exponential growth rate of each strain was compared to the WT using linear regression analysis. The growth rates of four of the transposon mutants were significantly lower than the WT: AO18Δ*nhaC*-T, AO18Δ*rfaI*-T, AO18Δ*hypo01*-T, and AO18Δ*abh*-T (p < 0.0001). Their isogenic mutant counterparts AO18Δ*nhaC*, AO18Δ*hypo01*, and AO18Δ*abh*, however, did not have slower growth rates. Instead, the isogenic mutants with significantly decreased growth rates were AO18Δ*nanM* and AO18Δ*rfaI* (p = 0.0045 and p < 0.0001, respectively), although the resulting concentrations of both strains in the stationary phase were similar to that of the WT. There was a slight recovery in growth in the complement AO18Δ*rfaI*-C but not AO18Δ*nanM*-C. Additionally, the complemented mutants AO18Δ*rfaY*-C (p < 0.0001), AO18Δ*nhaC*-C (p = 0.0026), AO18Δ*hypo01*-C (p = 0.0022), and AO18Δ*hypo14*-C (p < 0.0001) had significantly lower exponential growth rates than the WT.

#### Biofilm formation

**Fig 3:**
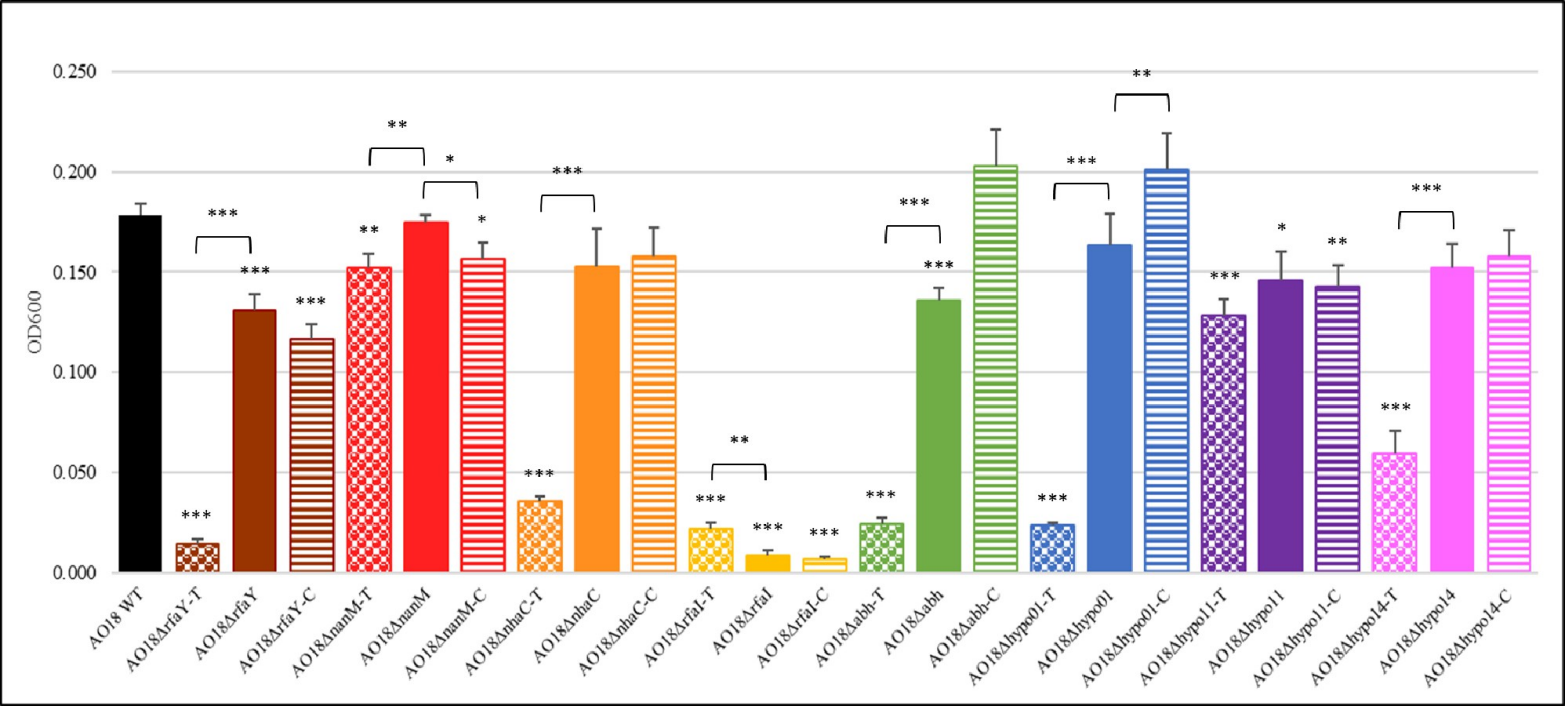
Biofilm production of all the strains tested. The biofilm formation ability of the transposon mutants, isogenic mutants, and complemented mutants were compared to that of the APEC O18 wild type (AO18 WT). Each test strain was grown statically for 24 h in M63 minimal medium and then evaluated for its biofilm formation ability using crystal violet assays. Each assay was performed with eight technical replicates on three separate days, and the absorbance data was averaged. Asterisks (*) above individual bars indicate a significant difference between that strain and AO18 WT, whereas asterisks above brackets joining two columns indicate significant difference between those two strains (*p < 0.05, **p < 0.01, ***p < 0.001).

To evaluate the importance of the selected genes in APEC biofilm formation, biofilm assays in M63 minimal medium were performed for AO18 WT, the isogenic mutants and complements, and their transposon mutant counterparts. The biofilm production of each strain is displayed in Fig 3. In these assays, the biofilm production of all transposon mutants is significantly decreased compared to that of the WT (p < 0.001). The following isogenic mutants also showed decreased biofilm production: AO18Δ*rfaY*, AO18Δ*rfaI*, AO18Δ*abh*, and AO18Δ*hypo11* (p < 0.001, 0.001, 0.001, 0.05, respectively), although AO18Δ*rfaY*, AO18Δ*rfaI*, and AO18Δ*abh* all had significantly increased biofilm production compared to their transposon counterparts (p < 0.001, 0.01, 0.001, respectively). AO18Δ*abh*-C was the only complement to make a full recovery of biofilm production back to that of the WT. AO18Δ*rfaY*-C, AO18Δ*rfaI*-C, and AO18Δ*hypo11*-C displayed no recovery. The complement AO18Δ*nanM*-C also had significantly decreased biofilm production compared to its isogenic mutant counterpart (p < 0.05).

#### Gene expression

**Fig 4:**
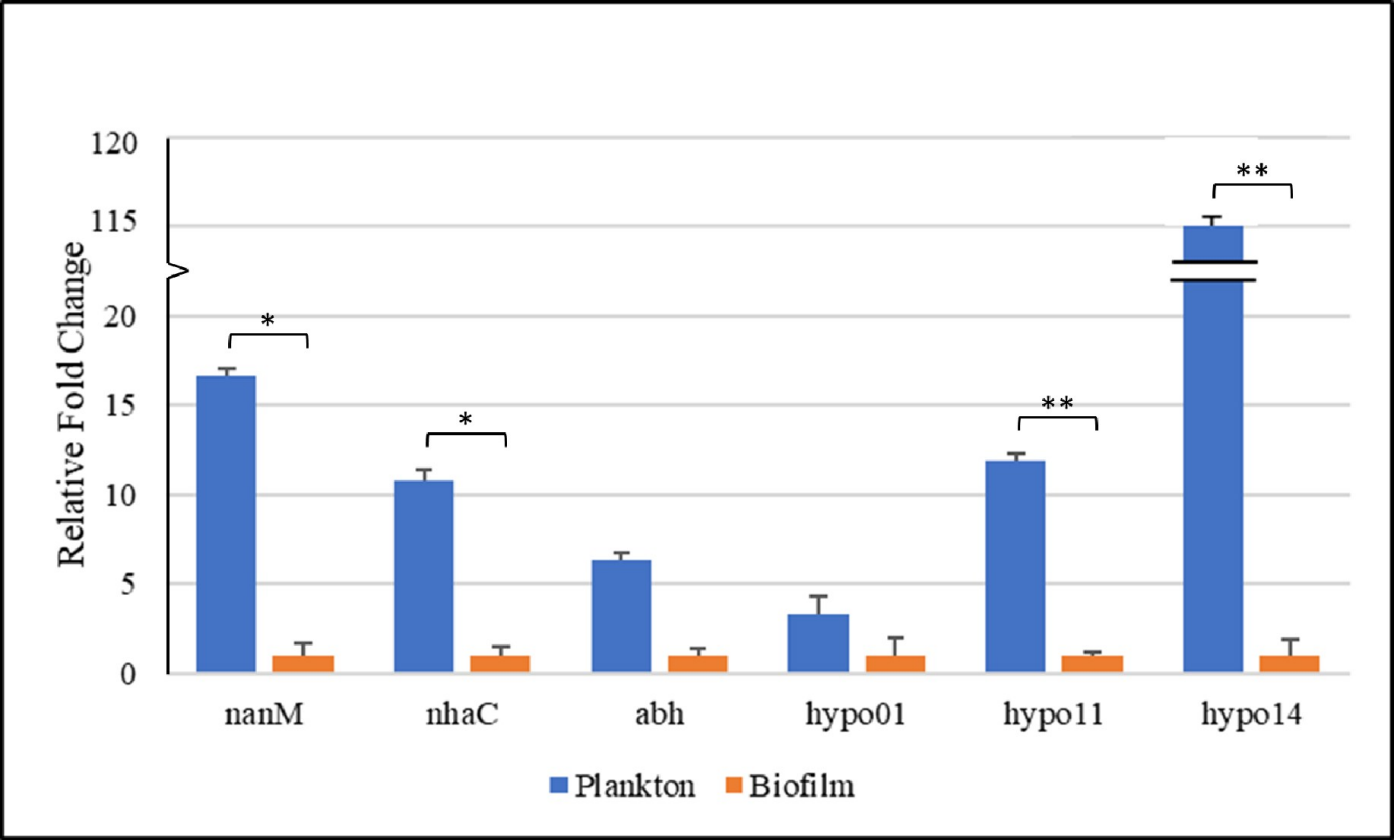
Comparison of expression of *nanM*, *nhaC*, *hypo01*, *abh*, *hypo11*, and *hypo14* during planktonic growth and mature biofilm phases. APEC O18 was grown until the exponential phase of growth in M63 minimal medium at 37°C to measure the expression of the planktonic cells. APEC O18 was grown statically for 24 h in M63 minimal medium at 37°C to form the mature biofilm. RNA from each phase of growth was extracted, purified, then reverse-transcribed into cDNA to read the expression of each gene. Expression was averaged among technical and biological triplicates and measured in fold change using the Livak method (*p < 0.01, **p < 0.001).

To better understand the behavior of APEC during planktonic growth and biofilm maturation, the expression of the target genes of interest was analyzed and compared in both phases. The 16S rRNA housekeeping gene was used as the endogenous control to normalize expression levels. The expression levels of *rfaY* and *rfaI* were unable to be analyzed due to a lack of primer binding to the sequence. As shown in Fig 4, there was a significant increase in expression of *nanM*, *nhaC*, *hypo11*, and *hypo14* during planktonic growth compared to the mature biofilm phase (fold changes: 16.61, 10.76, 11.88, and 115.74, respectively). Although *hypo01* and *abh* displayed numerically higher gene expression in the planktonic growth phase, there was no significant difference between planktonic growth and biofilm maturation expression levels.

## Discussion

APEC is a major problem for the poultry industry worldwide. It is a leading cause of morbidity and mortality, leading to substantial economic losses annually [1]. APEC also has the potential to cause disease in humans, including urinary tract infections and meningitis [31–33]. Biofilms, therefore, are an important virulence factor that contribute to the survival and persistence of APEC in the environment and the host. In order to better characterize the APEC biofilm, we used STM to identify APEC genes that contribute to biofilm formation in a well-characterized APEC strain.

From the 547 genes identified in this study, some previously described as being associated with biofilm formation were also found. Putative biofilm formation genes identified in this study included type I fimbriae genes *fimA*, *fimC*, *fimD*, *fimF*, *fimG*, *fimH*, and *fimI* and the transcriptional regulator *csgD*, which is a major biofilm regulator in both *E*. *coli* and *Salmonella* spp. [34–36]. Multiple outer membrane proteins were also found, including *pgaA*, *pgaB*, *ompA*, and both *envZ* and *ompR* of the EnvZ-OmpR two-component regulatory system, all of which have been shown to be involved in or regulate biofilm formation [37–39]. Other virulence genes were found also, such as hemolysin co‐regulated protein (Hcp) subunit *hcp1*. Hcp is a major component of the type VI secretion system, which is a virulence factor of APEC involved in adherence and pathogenicity and has been demonstrated to contribute to biofilm formation in APEC [29,40]. The finding of these biofilm-related genes validates the credibility of this study and allows us to move forward with our hypothesis. Since the disruption of these biofilm-related genes caused a decreased biofilm-formation ability in the APEC O18 mutants, it is reasonable to assume that the other genes found in our analysis may also be related to biofilm formation.

To test our hypothesis, isogenic mutants and complements were created for eight of the putative APEC biofilm genes (*rfaY*, *nanM*, *nhaC*, *rfaI*, *hypo01*, *abh*, *hypo11*, and *hypo14*). An isogenic mutant was not created for *rfaJ* due to its close proximity to *rfaY* and *rfaI* on the genome. Each mutant was evaluated for its role not only in the biofilm formation of APEC but also in its planktonic growth. Biofilm formation begins with the attachment of planktonic bacteria to a surface; therefore, the first step of biofilm formation relies on the fitness of planktonic bacteria. Slow-growing or otherwise-hindered bacteria will form biofilms more slowly or incompletely, resulting in poor biofilm structure, as seen in previous studies [41,42]. From this study, the deletion of three genes (*rfaY*, *abh*, and *hypo11*) were found to decrease biofilm production without decreasing the growth rate of APEC O18, while the deletion of *rfaI* induced both decreased planktonic growth and biofilm production.

A limitation of this study included the decrease in the growth rate and subsequent biofilm production of the complemented strains. This, however, may have been because the genes were re-inserted back on a plasmid, which may hinder full function. Previous studies have shown that plasmids increase the metabolic burden of the host bacteria, which may result in slower growth rates [43–46]. This observation may also explain the lack of recovery of the complemented strains in growth and biofilm formation, as the complemented strains themselves were impaired by the burden of the plasmid and displayed decreased growth when their deletion mutant counterparts did not.

In addition to the plasmid-bearing complemented strains, a limitation of this study was the inability to obtain results for expression of *rfaY* and *rfaI* in the planktonic growth and mature biofilm maturation stages as a result of inhibitors present. It is well-known that reverse transcriptase (RT) can have inhibitory effects on PCR [47–49]. RT inhibition may also be mediated through specific primer-template interactions [50,51], which would explain why the other primers could bind to the cDNA and exhibit gene expression. Although two-step qPCR should reduce the risk of RT interference, untreated cDNA, as was used in this study, may still harbor RT that will interfere with the qPCR reaction [47]. Therefore, the presence of RT from the untreated cDNA interacting with the *rfaY* and *rfaI* primers possibly resulted in the inhibition of primer binding to the template and the inability to analyze the transcripts.

Of the eight putative APEC biofilm formation genes analyzed in this study, four have been characterized (*rfaY*, *nanM*, *nhaC*, *rfaI*) and four are uncharacterized or identified as hypothetical proteins (*abh*, *hypo01*, *hypo11*, *hypo14*). *rfaY* and *rfaI*, also referred to as *waaY* and *waaI* or *waaO*, respectively, are both involved in the synthesis of the lipopolysaccharide (LPS), a major virulence determinant in *E*. *coli*. LPS constitutes a large portion of the outer membrane of Gram-negative bacteria and is composed of three structural domains: the lipid A endotoxin, the core oligosaccharide, and the O-antigen polysaccharide [52]. Additionally, LPS contributes to biofilm formation through bacterial adhesion, motility, and biosynthesis of colanic acid, the main exopolysaccharide of *E*. *coli* [8,53,54]. Both *rfaY* and *rfaI* are involved in the synthesis of the core oligosaccharide, with *rfaY* contributing to the inner core and *rfaI* to the outer core [55,56]. A previous study showed that the deletion of *rfaY* and *rfaI* in *E*. *coli* W3110 significantly decreased biofilm formation without decreasing the growth rate [57]. However, that previous study used LB broth to culture the strains for the growth curve compared to minimal medium in the current study, which may account for the difference in growth, as nutrient availability in media impacts bacterial growth rates [58].

Interestingly, when *in silico* PCR was performed, the primers used for the *rfaY* or *rfaI* of APEC O18 (*rfaY*_AO18_ and *rfaI*_AO18_) did not bind with *E*. *coli* W3110. A subsequent alignment of *rfaY*_AO18_ and *rfaI*_AO18_ to *E*. *coli* W3110 using Geneious Prime displayed significant variation between the *rfaY*_AO18_ and *rfaI*_AO18_ and those of *E*. *coli* W3110. Further alignments between *rfaY*_AO18_ and *rfaI*_AO18_ and other *E*. *coli* strains indicated that, although *rfaY* and *rfaI* genes were found in the *E*. *coli* K-12 strains examined, they also had significant variations from *rfaY*_AO18_ and *rfaI*_AO18_ (approximately 60% pairwise identity). However, 15 out of 17 of the APEC and other ExPEC strains analyzed harbored genes that fully align with *rfaY*_AO18_ and *rfaI*_AO18_, indicating that these genes may be specific to APEC or ExPEC. This may be due to the LPS core structure of each strain. Thus far, there are five characterized LPS core structures, denoted K-12, R1, R2, R3, and R4 [52]. APEC O18 and *E*. *coli* W3110 have different LPS core structures, classified as R1 and K-12, respectively. Research regarding the relationship between LPS core type and *E*. *coli* pathogenicity is limited; however, there may be a correlation between ExPEC and LPS core type based upon phylogenetic group and virulence genes [59–61]. According to these data and considering the significant difference in prevalence of *rfaY*_AO18_ and *rfaI*_AO18_ among the APEC (65% and 78%, respectively) and AFEC (14% and 31%, respectively) isolates analyzed by conventional PCR, the variations of *rfaY* and *rfaI* found in APEC O18 may be specialized for ExPEC.

Another characterized gene in this study was *nanM*, previously referred to as *yjhT*, which encodes a mutarotase of N-acetylneuraminic acid (Neu5Ac), a member of the sialic acid family [62]. *nanM* is a part of the *nan* operon and accelerates the spontaneous conversation of α-Neu5Ac to β-Neu5Ac, which is more stable and accessible for bacteria that obtain sialic acid from their environment [62]. Sialic acid is an energy source for *E*. *coli*, and it contributes to pathogen survival in the host and its ability to interact with host-cell surfaces [63,64]. Sialic acid regulation is also linked to the formation of biofilm-like intracellular bacterial communities in uropathogenic *E*. *coli* [65]. In this study, *nanM* was upregulated while growing in minimal medium compared to when the biofilm was mature. Since sialylation contributes to biofilm formation [66–68], *nanM* may have been upregulated as a result of the stressful environment in the minimal medium, converting α-Neu5Ac to β-Neu5Ac to prepare for biofilm formation. The nutrient-deficient environment in the minimal medium also may have upregulated *nanM* as a means to acquire more energy through sialic acid. In the phenotypic biofilm assay, there was no significant decrease in biofilm formation when *nanM* was deleted from APEC O18. This may be because of compensatory mechanisms in *E*. *coli* for sialic acid uptake, such as *nanS*, which is a putative sialate esterase that allows *E*. *coli* to grow on alternative sialic acids [69]. Although research on the role of sialic acid in APEC pathogenesis is limited, since APEC and uropathogenic *E*. *coli* (UPEC) are known to share virulence factors [32,33], it is reasonable to assume that sialic acid is important for APEC virulence also. This observation is supported by the prevalence of *nanM* in the APEC isolates (64%) compared to the AFEC isolates (15%) (Fig 1). Therefore, *nanM* may be a potential marker of APEC strains.

The final characterized gene was *nhaC*, which encodes the sodium:proton (Na^+^/H^+^) antiporter NhaC. *E*. *coli* is usually known to only carry Na^+^/H^+^ antiporters NhaA and NhaB: NhaA is the primary Na^+^/H^+^ antiporter responsible for conferring resistance to high levels of sodium and lithium, and NhaB is an alternate Na^+^/H^+^ antiporter when NhaA is not activated [70,71]. *nhaC* has been characterized in other species such as *Bacillus* spp. [72,73], and its insertion been found to functionally complement a *nhaA* deletion in *E*. *coli* [74]. *nhaC* is homologous to the *E*. *coli* gene *ibeT*, which encodes the putative transporter IbeT belonging to the Na+/H+ antiporter family [75]. *ibeT* resides on the *ibeRAT* operon on the GimA genomic island. It has been implicated in the adhesion of neonatal meningitis *E*. *coli* (NMEC) to brain microvascular endothelial cells (BMEC) both *in vitro* and *in vivo* and may also coordinately contribute to invasion with *ibeA* [76,77]. The upregulation of this gene during the growth phase in minimal medium may have been a mechanism of energy acquisition or preparation for attachment. However, neither *nhaC* nor *ibeT* have been directly implicated in biofilm formation to date, and the gene deletion did not significantly impair biofilm formation in APEC O18, although there was a numerical decrease observed. *ibeA*, on the other hand, has been associated with biofilm formation in APEC [78]. Therefore, similar to the role in BMEC invasion, *ibeT* may coordinately contribute to APEC biofilm formation with *ibeA*, although further research is required for confirmation.

The remaining four genes identified in this study were all uncharacterized. Full gene sequences were analyzed using BLASTN, but no characterized identical proteins could be found for the hypothetical proteins *hypo01*, *hypo11*, or *hypo14*. Interestingly, the expression of *hypo11* and *hypo14* in the planktonic phase of growth were both significantly higher than that observed during biofilm maturation. Even more noteworthy was that *hypo14* was expressed 115.74 times higher in the planktonic growth phase than in the biofilm maturation phase (Fig 4). Considering attachment factors are upregulated prior to bacterial attachment [8], *hypo11* and *hypo14* may potentially have involvement in adhesion. However, further characterization assays are required to elucidate the function of these genes.

The putative alpha-beta hydrolase (*abh*) returned one gene out of 696 identical protein accession numbers, resulting in the identification of carboxylesterase B *caeB*. Lun and Bishai [79] characterized the *caeB* paralog *caeA* in *Mycobacterium tuberculosis* and found that it encodes a cell wall-associated carboxylesterase that is required for *M*. *tuberculosis* virulence. However, *M*. *tuberculosis* is a Gram-positive species, and *E*. *coli* is Gram-negative. Goullet et al. [80] characterized some properties of *caeB* in *E*. *coli*, but the exact role of *caeB* in *E*. *coli* has yet to be identified. In the present study, the deletion of this putative *caeB* exhibited decreased biofilm formation in APEC O18 but did not decrease planktonic growth. In addition, its location on the APEC O18 genome is near genes encoding other hypothetical proteins and tail fiber assembly proteins. Therefore, *caeB* may play a role in adhesion or motility, but further studies are required to prove that the putative hydrolase in this study was *caeB* and what exact role it may play in APEC biofilm formation.

Transposon insertions can lead to incomplete disruptions in the genes or may block expression of downstream genes if inserted into an operon [81]. Polar effects of the transposon on the expression of adjacent genes may explain why the deletions of *nanM*, *nhaC* (*ibeT*), *hypo01*, and *hypo14* did not result in reduced biofilm formation in APEC O18 like that of the transposon mutants. For example, *ibeT* is on the same operon and downstream of *ibeA*. The transposon may have been inserted near the beginning of *ibeT* or between the two genes, therefore disturbing the function of *ibeA*, which is known to contribute to biofilm formation [78]. Similarly, the *nanM* is upstream of *nanS*, an alternate sialic acid enzyme. The transposon insertion in *nanM* could have also disrupted the function of *nanS*, severely inhibiting APEC O18’s ability to acquire sialic acid. The same logic applies to any other gene whose isogenic mutant does not match the properties of its transposon mutant counterpart.

In conclusion, eight genes have been found to be widespread in APEC and may contribute to APEC biofilm formation. This work lays the groundwork for further research, including how prevalent these genes are in APEC and how they contribute to APEC biofilm formation. In addition, four novel hypothetical proteins have been identified that appear to be widespread in APEC and may contribute to APEC biofilm formation. Further characterization assays are required to elucidate their functions and what exact roles they may play in *E*. *coli*.

## Supporting information

S1 Fig Functions of all putative biofilm formation genes found among the transposon mutants and the count of genes per function. The transposon mutants were sent for Sanger sequencing around the transposon insertion site, and the resulting sequences were analyzed using BLASTN. A total of 547 putative biofilm formation genes were identified, falling into 52 different categories of functions.
(TIF)

S2 Fig Growth curves of all the strains tested. The growth ability of the transposon mutants, isogenic mutants, and complemented mutants of *rfaY* (A), *nanM* (B), *nhaC* (C), *rfaI* (D), *abh* (E), *hypo01* (F), *hypo11* (G), and *hypo14* (H) were compared to the wild-type strain APEC O18 (AO18 WT). Each strain was grown in M63 minimal media for 12 h with shaking at 37°C, and the optical density at 595 nm was measured every 10 minutes. Growth curves were performed with eight technical replicates on three separate days, and the absorbance data was averaged and plotted against time to build the growth curves.
(TIF)

S1 Table Description of the genes used for the PCR prevalence analysis. (XLSX)
