# Supplementary figures and images for: Identification of novel genes involved in the biofilm formation process of Avian Pathogenic Escherichia coli (APEC)
Source: PLoS One. 2022 Dec 19;17(12):e0279206. doi: 10.1371/journal.pone.0279206 (PMC9762606; doi:10.1371/journal.pone.0279206)

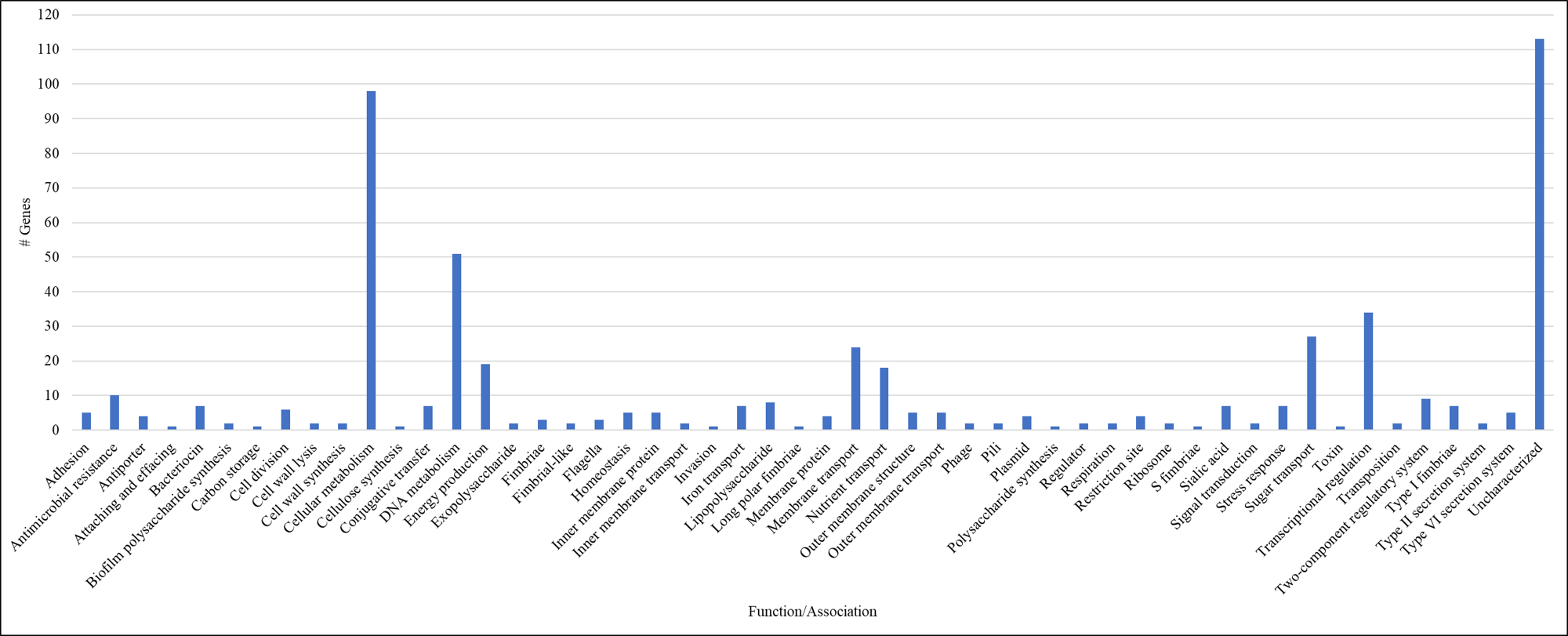

Supplement: S1 Fig — The transposon mutants were sent for Sanger sequencing around the transposon insertion site, and the resulting sequences were analyzed using BLASTN. A total of 547 putative biofilm formation genes were identified, falling into 52 different categories of functions. (TIF) [file pone.0279206.s001.tif]

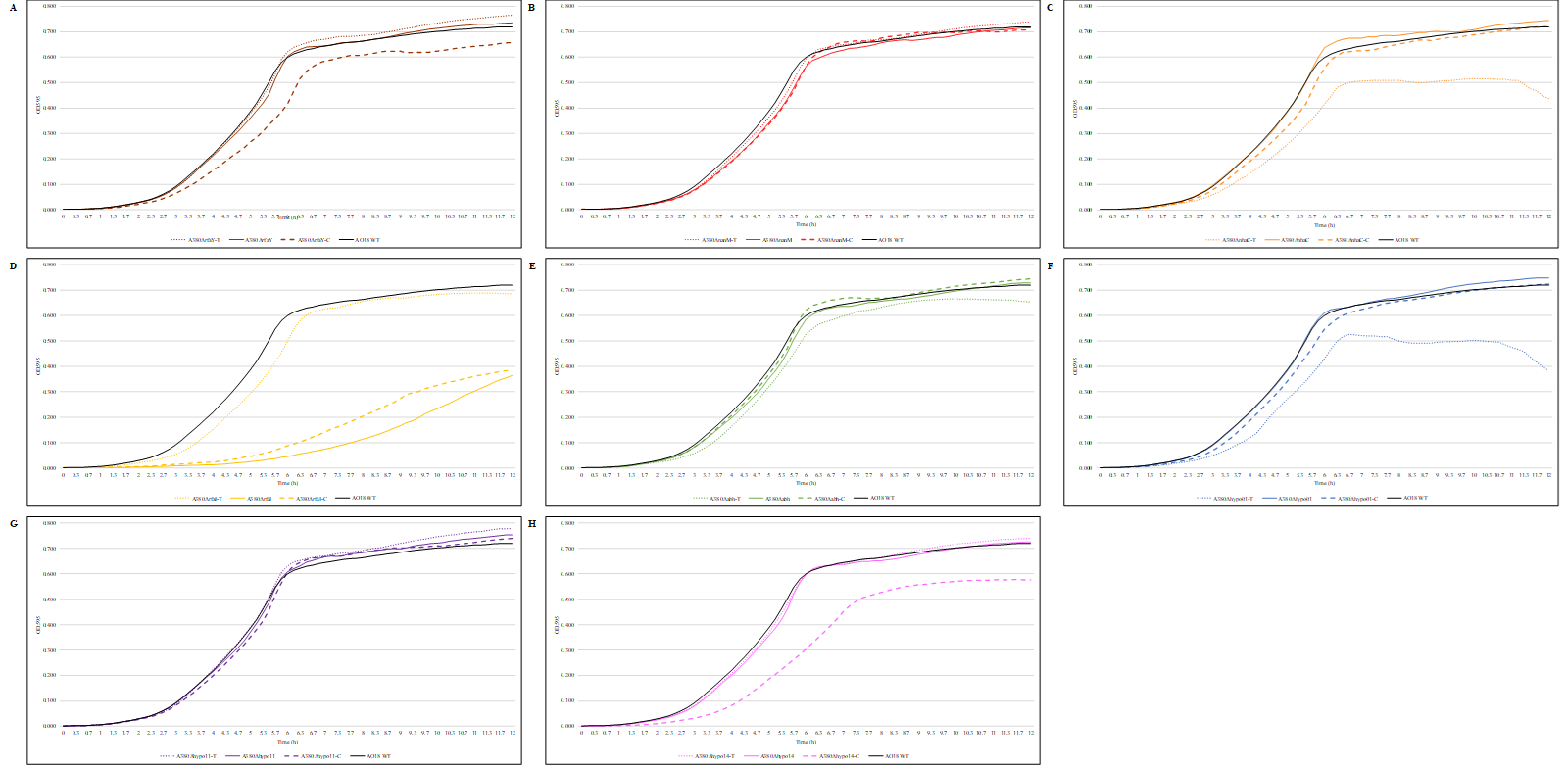

Supplement: S2 Fig — The growth ability of the transposon mutants, isogenic mutants, and complemented mutants of rfaY (A), nanM (B), nhaC (C), rfaI (D), abh (E), hypo01 (F), hypo11 (G), and hypo14 (H) were compared to the wild-type strain APEC O18 (AO18 WT). Each strain was grown in M63 minimal media for 12 h with shaking at 37°C, and the optical density at 595 nm was measured every 10 minutes. Growth curves were performed with eight technical replicates on three separate days, and the absorbance data was averaged and plotted against time to build the growth curves. (TIF) [file pone.0279206.s002.tif]
